# Survival of *BRCA1*/*BRCA2-*associated pT1 breast cancer patients, a cohort study

**DOI:** 10.1007/s10549-022-06608-1

**Published:** 2022-05-04

**Authors:** Mark van Barele, Amy Rieborn, Bernadette A. M. Heemskerk-Gerritsen, Inge-Marie Obdeijn, Linetta B. Koppert, Claudette E. Loo, Rob A. E. M. Tollenaar, Margreet G. E. M. Ausems, Irma van de Beek, Lieke P. V. Berger, Maaike de Boer, Liselot P. van Hest, C. Marleen Kets, Matti Rookus, Hebon, Marjanka K. Schmidt, Agnes Jager, Maartje J. Hooning

**Affiliations:** 1 Department of Medical Oncology, Erasmus MC Cancer Institute, University Medical Centre Rotterdam, Rotterdam, The Netherlands; 2 Department of Radiology, Erasmus MC Cancer Institute, University Medical Centre Rotterdam, Rotterdam, The Netherlands; 3 Department of Surgical Oncology, Erasmus MC Cancer Institute, University Medical Centre Rotterdam, Rotterdam, The Netherlands; 4 Department of Radiology, Division of Diagnostic Oncology, Netherlands Cancer Institute-Antoni van Leeuwenhoek Hospital, Amsterdam, The Netherlands; 5 Department of Surgery, Leiden University Medical Centre, Leiden, The Netherlands; 6 Division Laboratories, Pharmacy and Biomedical Genetics, Department of Genetics, University Medical Centre Utrecht, Utrecht, The Netherlands; 7 Department of Clinical Genetics, Amsterdam UMC, University of Amsterdam, Amsterdam, The Netherlands; 8 Department of Genetics, University of Groningen, University Medical Centre Groningen, Groningen, The Netherlands; 9 Division of Medical Oncology | Department of Internal Medicine, GROW-School of Oncology and Developmental Biology, Maastricht University Medical Centre, Maastricht, The Netherlands; 10 Department of Clinical Genetics, Amsterdam UMC, Vrije Universiteit Amsterdam, De Boelelaan 1117, Amsterdam, The Netherlands; 11 Department of Human Genetics, Radboud University Medical Centre, Nijmegen, The Netherlands; 12 Department of Epidemiology, Netherlands Cancer Institute-Antoni Van Leeuwenhoek Hospital, Amsterdam, The Netherlands; 13 Division of Molecular Pathology, Netherlands Cancer Institute, Amsterdam, The Netherlands

**Keywords:** *BRCA1*, *BRCA2*, Tumor size, Survival, Breast cancer

## Abstract

**Purpose:**

Intensive screening in *BRCA1/2* mutation carriers aims to improve breast cancer (BC) prognosis. Our aim is to clarify the prognostic impact of tumor size in *BRCA* mutation carriers with a pT1 BC, which is currently unclear. We are especially interested in differences between pT1a, pT1b, and pT1c regarding the prognosis of node-negative breast cancer, the effect of chemotherapy, and the prevalence of lymph node involvement.

**Methods:**

For this study, *BRCA1/2-*associated BC patients were selected from a nationwide cohort. Primary outcomes were 10-year overall survival (OS) per pT1a-b-c group and the effect of chemotherapy on prognosis of node-negative BC, using Kaplan–Meier and Cox models. Finally, we evaluated lymph node involvement per pT1a-b-c group.

**Results:**

963 women with pT1 *BRCA1/2-*associated BC diagnosed between 1990 and 2017 were included, of which 679 had pN0 BC. After a median follow-up of 10.5 years, 10-year OS in patients without chemotherapy was 77.1% in pT1cN0 and lower than for pT1aN0 (91.4%, *p* = 0.119) and pT1bN0 (90.8%, *p* = 0.024). OS was better with than without chemotherapy for pT1cN0 (91.6% vs. 77.1%, *p* = 0.001; hazard ratio (HR) 0.56, 95% confidence interval (CI): 0.21–1.48). Lymph node involvement was 24.9% in pT1c, 18.8% in pT1b, and 8.6% in pT1a.

**Conclusion:**

Smaller tumor size is associated with better OS and less lymph node involvement in pT1 *BRCA1/2*-associated BC patients. The results suggest that early detection in *BRCA1/2* mutation carriers of pT1a/b BC may reduce mortality and the need for systemic therapy.

**Supplementary Information:**

The online version of this article contains supplementary material available (10.1007/s10549-022-06608-1.

## Introduction

Women carrying a pathogenic germline *BRCA1* or *BRCA2* mutation have life-time breast cancer (BC) risks up to 75%, and are often diagnosed with BC at a relatively young age [1]. Therefore, *BRCA1/2* mutation carriers may opt for bilateral prophylactic mastectomy, reducing BC risk to almost zero, or participate in a tailored BC screening program [2]. The purpose of screening is to find BC in an early stage with excellent prognosis, preferably without the necessity of endocrine therapy or (neo)adjuvant chemotherapy. A prerequisite for the latter is the absence of nodal involvement.

In the general population, randomized controlled trials (RCT) of mammography screening demonstrated a reduction in BC mortality [3]. Consequently, the general consensus is that actively trying to find BC at an early stage is effective, and a nationwide screening program for women aged 50–75 years was implemented in many European countries. The basis for effective screening lies in the fact that sporadic BC patients with a small node-negative tumor have an excellent prognosis [4]. Tumor size and lymph node involvement are positively correlated and both are independent predictors for BC-related mortality [5, 6]. These findings reinforce the rationale behind population-wide screening programs. For *BRCA1/2* mutation carriers, however, no trials investigating screening exist, and screening efficacy is only presumed to be similar to that of the general population [7]. At the age of 25—the recommended age to start BC screening for *BRCA1/2* mutation carriers—breast density is higher than for women in regular screening programs, possibly affecting mammography efficacy. Therefore, and to avoid radiation exposure, MRI is already the modality of choice in young women. Additionally, debate is still ongoing whether the association between tumor size and outcome is as strongly present in *BRCA1/2-*associated BC [8–11]. Moreover, the correlation between tumor size and lymph node involvement in *BRCA1* mutation carriers has been reported to be weaker than for sporadic BC or *BRCA2*-associated BC [12]. Together, these findings imply uncertainty regarding survival benefit from BC screening in *BRCA1/2* mutation carriers.

Screening programs tailored to *BRCA1/2* mutation carriers have previously been investigated, but so far no studies could directly demonstrate survival benefit from BC screening [13, 14]. Given that data on the prognostic value of tumor size in *BRCA1/2* mutation carriers are currently lacking, the purpose of the current study is to determine the prognostic value of this tumor characteristic within a population of *BRCA1/2* mutation carriers. First, we evaluate whether tumor size, categorized as pT1a (0.1–0.5 cm), pT1b (> 0.5–1.0 cm), and pT1c (> 1.0–2.0 cm) according to the *TNM classification of malignant tumors*, UICC (TNM), is a good prognostic factor of survival in pT1N0 *BRCA1/2-*associated BC patients who did not receive chemotherapy. We are especially interested in the natural course of disease without chemotherapy and the possibilities to omit this treatment modality for patients with small node-negative tumors, for chemotherapy can severely impact quality of life. Second, we evaluate the effect of chemotherapy in *BRCA1/2-*associated BC. Third, as lymph node involvement has consequences for both treatment (regardless of tumor size), and prognosis in the general BC population, we evaluate the association between tumor size and lymph node status in *BRCA1/2-*associated BC patients.

## Patients and methods

Eligible participants were retrieved from the ongoing national HEBON (Hereditary Breast and Ovarian Cancer Research Netherlands) study cohort. In the HEBON study, members of families with pathogenic germline *BRCA1/2* mutations were identified through the departments of Clinical Genetics of the eight Dutch academic medical centers and the Netherlands Cancer Institute. All participating centers’ Medical Ethics Committees approved the study. Written informed consent was acquired from each participating woman, or a close relative or proxy for deceased individuals. Relevant data on patient characteristics, cancer diagnosis, tumor characteristics, and preventive strategies were retrieved and updated through linkage with the Dutch National Cancer Registry and the national pathology database, and from medical files and questionnaires. Therefore, this registry is a mixture of both retrospective data collection and prospective follow-up [15]. The latest follow-up date used in the current study was December 31, 2017.

We included only women carrying a proven pathogenic germline *BRCA1* or *BRCA2* mutation via testing, and diagnosed with pT1a, pT1b, or pT1c breast cancer between 1 January 1990 and 1 January 2017 (regardless of N-status). BC was diagnosed either before or after confirmation of *BRCA1/2* mutation carrier status. Patients were excluded if they had distant metastases at BC diagnosis, had received neoadjuvant chemotherapy (i.e., lacking pT assessment), or had any other primary tumor prior to BC diagnosis (except cervical intraepithelial neoplasia (CIN), skin basal cell carcinoma (BCC) or skin squamous cell carcinoma (SCC)). Further reasons for exclusion were insufficient data regarding tumor size, vital status, or date of death.

### Data collection

We retrieved dates of birth, BC diagnosis, DNA test, and death. We retrieved information on TNM classification current at the time of diagnosis, mode of detection, estrogen receptor (ER) status, progesterone receptor (PR) status, Human Epidermal Growth Factor Receptor 2 (HER2)-status, type of surgery, chemotherapy, HER2-targeted treatment, endocrine treatment, radiotherapy, risk-reducing salpingo-oophorectomy (RRSO), risk-reducing mastectomy (RRM), *BRCA* mutation status, and vital status. All reported tumor stadia were histologically determined. ER-status and PR-status were defined positive if immunohistochemical staining showed 10% or more of the tumor cells positive for the receptor. The HER2-status was positive if the immunohistochemical staining was 3 + , or 2 + with a positive in-situ hybridization (ISH) test.

### Statistical analyses

The primary outcome was 10-year overall survival (OS) from date of BC diagnosis. To allow for prevalent cases at date of genetic testing, we applied left truncation in the survival analyses [16]. Time at risk therefore started at date of BC diagnosis or of DNA test result, whichever came last, and ended at date of death or date of censoring event. This does not change the moment 10-year OS is evaluated, but modifies the time at risk during this 10-year period. Censoring events were any non-breast primary malignancy (with the exception of CIN, BCC and SCC) or the last date of follow-up.

We used Kaplan–Meier survival analysis and the Log-Rank test to compare unadjusted OS curves of the pT1aN0, pT1bN0, and pT1cN0 subgroups not receiving chemotherapy, and of patients receiving chemotherapy vs no chemotherapy within the pT1N0 subgroups. Cox proportional hazards regression models were used to calculate hazard ratios (HR) and 95% confidence intervals (95% CI) for OS. We applied a subject matter knowledge-based model-building strategy, as we were interested in the etiological impact of tumor size on prognosis, and not in prediction. The following relevant clinical or pathological variables were included in the multivariable model: mutational status, ER-status, HER2-status, chemotherapy, endocrine therapy, RRSO, year of diagnosis, and age at diagnosis.

Finally, we also investigated the percentage of lymph node involvement at time of BC diagnosis, stratified by tumor size. As early detection is a major determinant of lymph node involvement, we further stratified by screening status. Missing data for screening were imputed by using the timing of DNA test result respective of BC diagnosis, as women with a DNA test result before BC diagnosis are very likely to participate in screening [17].

Descriptive statistics are shown as proportions or median and range. We used Pearson’s χ^2^ test to compare categorical variables and the Kruskal–Wallis test for continuous variables. All *p* values are two-sided.

Statistical analyses were performed using STATA 15.1.

Because of missing data for several important covariables (such as the HER2-status), we implemented a multiple imputation model to allow for all variables to be included in the multivariable Cox models without losing observations. The imputation model was built in STATA 15.1, see supplemental materials (Online Resource 1).

The models were checked for interactions between the main variable of interest with all other included variables. We tested the proportional hazards assumption through the addition of time-varying effects to the model, using a cut-off of *p* < 0.05 for the time-varying term.

## Results

### Patient characteristics

**Fig. 1:**
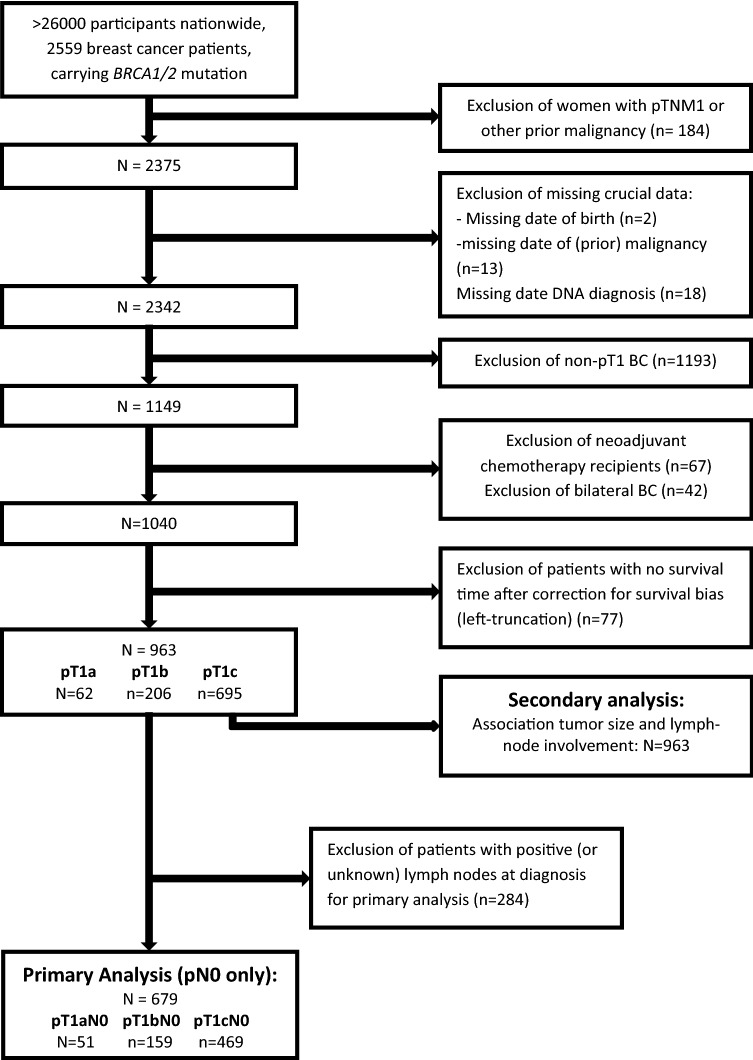
Flowchart of patient selection. *BC* breast cancer, *pTNM* pathological assessment of tumor size, lymph nodes and metastasis (pT1a = 0.1–0.5 cm, pT1b ≥ 0.5–1.0 cm, pT1c ≥ 1.0–2.0 cm)

We included 963 women with primary *BRCA1/2-*associated pT1 BC (Fig. 1). A total of 679 women had lymph node-negative disease at diagnosis (70.5%), of whom 51 (7.5%) were diagnosed with a pT1a tumor, 159 (23.4%) with a pT1b tumor, and 469 (69.1%) with a pT1c tumor. A full overview of patient, tumor, and treatment characteristics of the node-negative patients is provided in Table 1. Median follow-up time of the node-negative cohort was 10.5 years, and median age at BC diagnosis was 42.6 years. Patients with a pT1cN0 tumor received chemotherapy in 60.1% of cases, compared to 34.0% of pT1bN0 patients and 15.7% of pT1aN0 patients. *BRCA1* mutation carriers more often had a larger tumor than *BRCA2* mutation carriers (pT1c 71.6% vs 63.3%, *p* = 0.030, data not shown) and more often had an ER-negative tumor (79.7% vs 26.4%, *p* < 0.001, data not shown).

**Table 1:** Comparison of patient and tumor characteristics of patients diagnosed with node-negative pT1a, pT1b and pT1c BC

*N* = 679 total	pT1aN0 *N* = 51 (7.5%)	pT1bN0 *N* = 159 (23.4%)	pT1cN0 *N* = 469 (69.1%)	p-value
Follow-up time in years, median (range)	8.5 (1.3–15.3)	9.4 (0.6–27.5)	11.0 (0.5–27.6)	< 0.001
Age at diagnosis, median years (range)	44.6 (29.6–75.0)	44.9 (20.6–72.5)	42.0 (22.0–78.8)	0.07
Year of diagnosis, median (range)	2008 (1992–2015)	2006 (1990–2016)	2004 (1990–2017)	< 0.001
*Year of diagnosis, 5-year categories*				< 0.001
1990–1994	3 (5.9)	12 (7.6)	61 (13.0)	
1995–1999	0 (0.0)	21 (13.2)	87 (18.6)	
2000–2004	10 (19.6)	34 (21.4)	111 (23.7)	
2005–2009	22 (43.1)	43 (27.0)	133 (28.4)	
2010–2014	13 (25.5)	41 (25.8)	70 (14.9)	
2015–2017	3 (5.9)	8 (5.0)	7 (1.5)	
*BRCA mutation*				0.03
BRCA1	28 (54.9)	105 (66.0)	336 (71.6)	
BRCA2	23 (45.1)	54 (34.0)	133 (28.4)	
*Timing of BRCA DNA diagnosis*				< 0.001
After BC diagnosis	23 (45.1)	92 (57.9)	381 (81.2)	
Before BC diagnosis	28 (54.9)	67 (42.1)	88 (18.8)	
*Tumor grade*				< 0.001
1	6 (12.0)	10 (6.9)	18 (4.4)	
2	21 (42.0)	47 (32.4)	78 (19.1)	
3	23 (46.0)	88 (60.7)	313 (76.5)	
Unknown	1	14	60	
*ER-status*				0.004
ER+	21 (47.7)	63 (46.3)	123 (32.1)	
ER−	23 (52.3)	73 (53.7)	260 (67.9)	
Unknown	7	23	86	
*PR-status*				0.03
PR +	14 (31.1)	47 (36.2)	91 (24.5)	
PR -	31 (68.9)	83 (63.8)	280 (75.5)	
Unknown	6	29	98	
*HER2-status*				0.30
HER2 +	5 (14.7)	7 (7.1)	18 (7.3)	
HER2-	29 (85.3)	92 (92.9)	229 (92.7)	
Unknown	17	60	222	
*Type of surgery*				0.09
No surgery	0 (0.0)	0 (0.0)	0 (0.0)	
Lumpectomy	19 (38.0)	75 (49.3)	243 (53.6)	
Mastectomy	31 (62.0)	77 (50.7)	210 (46.4)	
Unknown	1	7	16	
*Radiotherapy received*				0.02
Yes	18 (35.3)	70 (44.6)	247 (53.4)	
No	33 (64.7)	87 (55.4)	216 (46.6)	
Unknown	0	2	6	
*Chemotherapy*				< 0.001
Yes	8 (15.7)	54 (34.0)	282 (60.1)	
No	43 (84.3)	105 (66.0)	187 (39.9)	
*Chemotherapy regimen*				0.06
CMF	0	1 (3)	13 (7)	
Anthracyclines	2 (40)	21 (57)	127 (70)	
Anthracyclines + Taxanes	3 (60)	12 (32)	38 (21)	
Other (e.g., only taxanes, platina-based)	0	3 (8)	3 (2)	
Unknown	3	17	101	
*Endocrine therapy*				0.009
Yes	3 (5.9)	20 (12.7)	93 (20.1)	
No	48 (94.1)	137 (87.3)	370 (79.9)	
Unknown	0	2	6	
*HER2-targeted therapy*				
Yes	1 (2.0)	4 (2.6)	10 (2.2)	0.95
No	50 (98.0)	153 (97.4)	453 (97.8)	
Unknown	0	2	6	
*RRM*				0.59
Contra-/bilateral RRM	29 (56.9)	94 (59.5)	263 (56.1)	
Ipsilateral RRM	3 (5.9)	13 (8.2)	26 (5.5)	
No RRM	19 (37.3)	51 (32.3)	180 (38.4)	
Unknown	0	1	0	
*Timing RRSO*				< 0.001
No RRSO	10 (19.6)	20 (12.9)	79 (16.8)	
Before BC	16 (31.4)	45 (29.0)	53 (11.3)	
After BC	25 (49.0)	89 (57.4)	337 (71.9)	
At the same time	0 (0.0)	1 (0.6)	0 (0.0)	

BC, breast cancer; ER, estrogen receptor; PR, progesterone receptor; HER2, Human epithelial growth factor receptor 2 (*ERBB2*); CMF, Cyclophosphamide, Methotrexate and 5-Fluorouracil; RRM, risk-reducing mastectomy; RRSO, risk-reducing salpingo-oophorectomy

### Ten-year overall survival in pT1N0 breast cancer

**Fig. 2:**
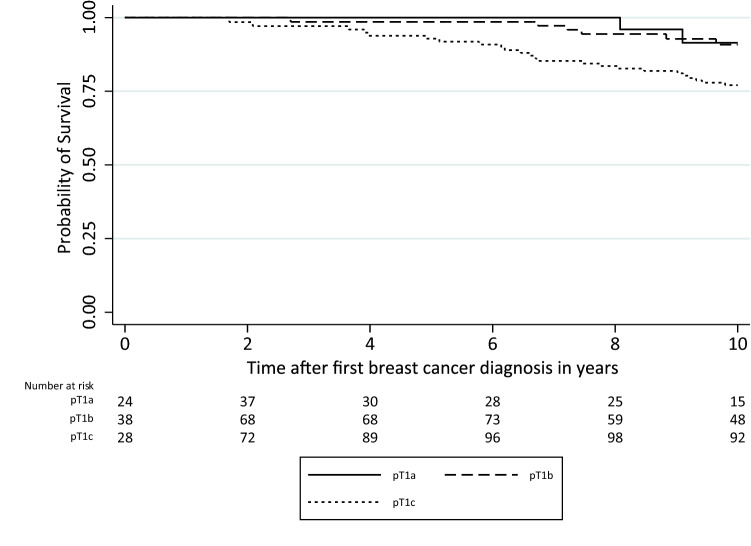
Kaplan–Meier of overall survival in node-negative pT1 (≤ 2.0 cm) breast cancer patients who did not receive chemotherapy. Abbreviations: pT, pathological tumor assessment (pT1a = 0.1–0.5 cm, pT1b ≥ 0.5–1.0 cm, pT1c =  > 1.0–2.0 cm)

In women who did not receive chemotherapy, 10-year OS was 77.1% for pT1c patients and lower than for pT1a (91.4%; *p* = 0.08) and pT1b (90.8%; *p* = 0.02) patients (Table 2; Fig. 2). When stratified by *BRCA1/2* mutation, a similar OS was seen for the different tumor sizes, except for pT1b (97.4% 10-year OS in *BRCA1* vs 82.8% in *BRCA2,* *p* = 0.049)(Table 2). Among pT1N0 chemotherapy recipients (*BRCA1* and *BRCA2* combined), 10-year OS of pT1a patients (69.4%) was worse than for pT1b (100%, *p* < 0.001) and pT1c patients (91.6%, *p* = 0.04)(Table 2).

**Table 2:** Ten-year overall survival of node-negative pT1 breast cancer

		pT1a *N* = 51 (7.5%)	pT1b *N* = 159 (23.4%)	pT1c *N* = 469 (69.1%)	Overall comparison
Overall node-negative population (*n* = 679)					
Number of deaths (%)		4 (7.8)	6 (3.8)	42 (9.0)	0.11
10-year overall survival		87.5%	93.4%	86.2%	0.12^a^
Number of deaths per subgroup (%)	Chemotherapy No chemotherapy	2/8 (25.0) 2/43 (4.7)	0/54 (/00) 6/105 (5.7)	18/282 (6.4) 24/187 (12.8)	0.01 0.07
10-year overall survival	Chemotherapy No chemotherapy Direct comparison	69.4% 91.4% *p* = 0.008	100% 90.8% *p* = 0.11	91.6% 77.1% *p* < 0.001	0.01^b^ 0.02^c^
Subgroup analysis: pN0 patients without the use of adjuvant chemotherapy, stratified by mutational status (*n* = 335)					
Number of deaths per subgroup (%)	*BRCA1* *BRCA2*	1/21 (4.8) 1/22 (4.5)	1/62 (1.6) 5/43 (11.6)	13/114 (11.4) 11/73 (15.1)	0.06 0.42
10-year overall survival	*BRCA1* *BRCA2* Direct comparison	90.9% 92.3% *p* = 0.99	97.4% 82.8% *p* = 0.049	76.9% 74.6% *p* = 0.67	0.02 0.34

^a^Individual comparisons: pT1a vs. pT1b, *p* = 0.25; pT1a vs. pT1c, *p* = 0.70; pT1b vs. pT1c, *p* = 0.038

^b^Individual comparisons: pT1a vs. pT1b, *p* < 0.001; pT1a vs. pT1c, *p* = 0.04; pT1b vs. pT1c, *p* = 0.07

^c^Individual comparisons: pT1a vs. pT1b, *p* = 0.83; pT1a vs. pT1c, *p* = 0.08; pT1b vs. pT1c, *p* = 0.02

For pT1cN0 and pT1bN0 patients, OS was better with chemotherapy than without (91.6% vs. 77.1%, *p* = 0.001, and 100% vs 90.8%, *p* = 0.11, respectively). In pT1aN0 patients, OS was worse for those receiving chemotherapy than for those without (69.4% vs 91.4%, *p* = 0.008)(Table 2).

### Adjusted survival analyses

For node-negative patients not receiving chemotherapy, adjusted HRs for overall mortality of 0.71 (95% CI 0.14–3.51) for pT1a and of 0.36 (95% CI 0.14–0.94) for pT1b were found when compared to pT1c patients (Table 3). Among pT1cN0 patients, chemotherapy compared to none revealed an adjusted HR for overall mortality of 0.56 (95% CI 0.21–1.48)(Table 3).

**Table 3:** Hazard ratios for 10-year overall mortality from univariable and multivariable Cox proportional hazards models

	Node-negative, no chemotherapy (*n* = 335)		Node-negative, pT1c (*n* = 469)	
	Univariable model HR (95% CI)	Multivariable model^a^ HR (95% CI)	Univariable model HR (95% CI)	Multivariable model^a^ HR (95% CI)
*Tumor size*			n/a	n/a
pT1c	1.00 (ref)	1.00 (ref)		
pT1a	0.30 (0.07–1.25)	0.71 (0.14–3.51)		
PT1b	0.36 (0.15–0.89)	0.36 (0.14–0.94)		
Chemotherapy, yes vs no	n/a	n/a	0.33 (95% CI 0.18–0.62)	0.56 (0.21–1.48)

^a^Adjusted for age, year of diagnosis, type of *BRCA* mutation, estrogen receptor status, HER2-status, grade, endocrine therapy, and risk-reducing salpingo-oophorectomy (as time-dependent variable)

### Lymph node involvement

**Fig. 3:**
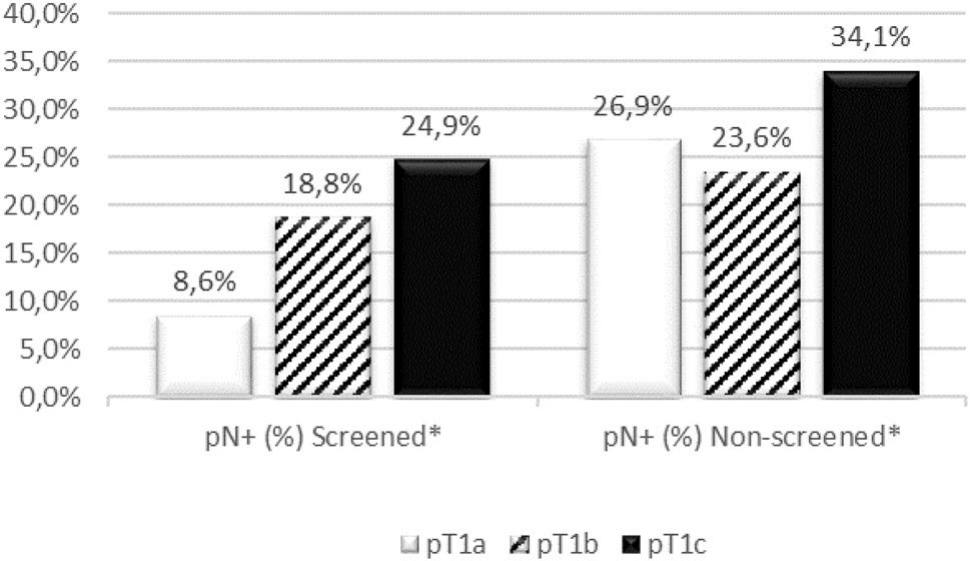
Percentage lymph node involvement in screened and non-screened patients. *known to be screened or not, missing values imputed based on DNA diagnosis (see methods for details). Abbreviations: pN, pathological lymph node assessment (pN ± lymph nodes are tumor-positive); pT, pathological tumor assessment (pT1a = 0.1–0.5 cm, pT1b ≥ 0.5–1.0 cm, pT1c ≥ 1.0–2.0 cm)

In the total pT1 population (*n* = 963), 28.5% of patients had lymph node involvement at diagnosis. The proportion with positive lymph nodes increased with larger tumor size at diagnosis: 16.4% for pT1a, 20.9% for pT1b, and 32.1% for pT1c (data not shown). In the screened population (*n* = 322, 33.4%), we found 20.9% lymph node involvement (8.6% for pT1a, 18.8% for pT1b, and 24.9% for pT1c; Fig. 3). When evaluated separately, in both *BRCA1* and *BRCA2* mutation carriers, pT1b and pT1c patients more often had lymph node involvement at diagnosis than pT1a patients (Online Resource 1). The highest proportion of lymph node involvement was found in *BRCA2* mutation carriers with a pT1c tumor (Online Resource 1).

## Discussion

The results of our study showed that, as is the case for non-hereditary BC, smaller tumor size was associated with improved OS in pT1N0M0 *BRCA1/2-*associated BC patients. We found a strong indication that adjuvant chemotherapy improves survival for patients with a pT1cN0 tumor. Twenty-nine percent of the total population had lymph node involvement, its prevalence increasing with larger tumor size at diagnosis.

Our results in pT1N0 BC patients contradict those of previous studies. Narod et al. observed no significant difference in OS between *BRCA1* mutation carriers with node-negative tumors of 0.1–1 cm and of 1–2 cm (adjusted HR 1.39, 95% CI 0.67–2.90) [8]. Similarly, Huzarski et al. found no significant association for *BRCA1* mutation carriers between tumor size and OS within the pT1 subgroup (adjusted HR 0.92, 95% CI 0.33–2.57, for tumors > 1 cm) [9]. This study included only 40 patients with a pT1a or pT1b tumor, limiting the probability of finding a difference. Another explanation for the different results, however, is that Huzarski et al. included patients treated with neo-adjuvant chemotherapy (66 out of 233), thereby potentially misclassifying clinically larger tumors as pT1, resulting in HRs closer to 1.00.

Systemic therapy can improve outcome of pT1 sporadic BC, although only of selected subgroups, and the effect might be small [18, 19]. A study among pT1 node-negative *BRCA1*-associated BC patients suggested that patients who did not receive chemotherapy appeared to have worse overall survival (OS) than patients who did receive chemotherapy [8]. While the effect is both small and limited to specific subgroups in sporadic patients, *BRCA1* mutation carriers with pT1N0 BC appear to benefit more from chemotherapy. One of the explanations for this might be that BC in *BRCA1* mutation carriers more often is of the triple-negative or basal-like phenotype, and of higher histologic grade [20, 21]. Therefore, more patients with pT1 *BRCA1*-associated BC could benefit from chemotherapy than previously assumed, even when detected by screening in an early stage. While we cannot demonstrate a direct benefit of chemotherapy, we did see that those treated with chemotherapy had better OS than those not treated with chemotherapy, with the exception of women with a pT1a tumor. Considering the small proportion of pT1aN0 patients receiving chemotherapy, this finding may simply be due to chance. Caution is warranted however, as we do not know the reason why physicians chose to advise adjuvant chemotherapy for some, but not all patients. Consequently, the chemotherapy and non-chemotherapy groups may not be comparable in terms of prognosis.

Despite screening, still 8.6% of pT1a, 18.8% of pT1b, and 24.9% of pT1c BC patients had positive nodes at diagnosis. This appears to be similar to the general population [22–24], but is higher than recently reported based on SEER data from 2010 to 2014. In that study, the overall lymph node positivity ranged from 1.4% for pT1a to 6.0% for pT1c [25]. We can only speculate on explanations for this remarkable difference. Possibly, as the latter study describes more recent data than ours, a stage-shift may have occurred with improving imaging techniques over the years, resulting in earlier BC detection with more smaller and node-negative tumors. Another explanation may be that *BRCA*-associated breast tumors show a different biological background than tumors among the general population (as used in the study by Zhao et al. [25]). Indeed, we observed especially among *BRCA2* mutation carriers high rates of lymph node involvement. The positive association between lymph node involvement and tumor size appears to be stronger in *BRCA2* mutation carriers than in *BRCA1* mutation carriers. Earlier work by Foulkes et al. also showed a significant positive correlation between tumor size and lymph node involvement for *BRCA2-*associated BC. However, they observed no clear association among *BRCA1*-associated BC patients [12]. This may have been due to smaller sample sizes. The observation that lymph node involvement is more frequent in *BRCA2* mutation carriers may be the result of tumor biology. Numerous reports suggest that hormone receptor-positive BC is indeed more likely to spread to the lymph nodes than triple-negative BC [26–29]. In our population, 78.8% of the *BRCA2* mutation carriers were diagnosed with a hormone receptor-positive BC, compared to only 24.2% of the *BRCA1* mutation carriers (*p* < 0.001).

One of the strengths of our study was the ability to assemble a large cohort of *BRCA1/2-*associated BC patients, with a relatively long follow-up. Furthermore, studies directly investigating the impact of screening usually have to deal with length–time bias and lead-time bias [30]. Because we primarily investigated the effect of tumor size (and not screening) on survival, these biases are unlikely to have affected our results. Lead-time bias could in theory still apply if there is a large screening differential among the tumor size groups. However, 10-year OS of pT1cN0 was worse compared to pT1bN0 and comparable to pT1aN0, irrespective of screening (89.5% compared to 96.8% and 90.7% with screening, 85.4% compared to 90.1% and 84.6% without screening, data not shown). Although we do see a (non-significant) change in OS with increasing tumor size, the absolute OS differences between pT1 subgroups remain fairly constant in both screened and unscreened patients, and therefore do not indicate the presence of (meaningful) bias. It should be noted that screening efficacy could be influenced by the fact that RRSO may decrease breast density. In the current cohort, uptake of RRSO before BC diagnosis was higher in pT1a/pT1b patients than in pT1c patients. Denser breast tissue may have delayed diagnosis of BC in the pT1c group. Possibly, if pT1c patients had undergone RRSO before a BC diagnosis, they might have been diagnosed with a pT1a or pT1b tumor instead.

A minor limitation is that we did not have a cohort of sporadic BC cases to directly compare our results with. Previously, several studies compared OS or breast cancer-specific survival (BCSS) between *BRCA1/2-*associated and sporadic BC. It is currently unclear whether overall survival is worse for *BRCA* mutation carriers, but if assumed to be the case, this may be largely due to the higher incidence of triple-negative BC (TNBC) within the *BRCA1* population as well as the potential for survival bias [31–34]. Further, a high incidence of TNBC in especially *BRCA1* mutation carriers may result in another complication. We used left-truncation to minimize survival bias from BC patients who were tested for *BRCA* mutation after their BC diagnosis. However, because the hazard for death is especially high in the first few years after diagnosis of TNBC, selection of favorable TNBC cases may still occur when the interval between BC diagnosis and testing is long. While this did not seem to be the case for our cohort (median time to testing 1.6 years, data not shown), one can never truly know which patients are missing due to selection bias, and the survival rates reported here may be an overestimation. However, comparing survival of only those who were tested for a BRCA mutation before breast cancer diagnosis (prospective analysis), we found that the 10-year survival rates are 100% for pT1aN0, 98.3% for pT1b, and 89.2% for pT1c. All are higher than what we found for the whole cohort (Table 2: 87.5%, 93.4% and 86.2% for pT1aN0, pT1bN0, and pT1cN0 respectively). In our opinion, this makes it less likely that survival is overestimated for newly diagnosed carriers.

This also suggests that *BRCA1* and *BRCA2* mutation carriers should ideally be analyzed separately, due to their tumor’s biological differences. However, despite having a much larger population of *BRCA* mutation carriers than any study before, this would still result in subgroups too small to draw reasonable conclusions. Instead, we opted to adjust the multivariable models for these biological differences, as well mutation status (i.e., *BRCA1* or *BRCA2*). A more important limitation was the small number of pT1a patients, making it difficult to draw useful conclusions about their prognosis. Combining the pT1a and pT1b groups into a 0.1–1.0 cm category could improve power, but would only allow for a generalized clinical application. When combined, we find 10-year survival rates of 91.9% for pT ≤ 1 cm (T1aN0/T1bN0) and 86.2% for pT > 1 cm (*p* = 0.02). For chemotherapy recipients, 10-year survival rates were 96.0% for pT ≤ 1 cm and 91.6% for pT > 1 cm. Among patients not receiving chemotherapy, 10-year survival was 91.0% for pT ≤ 1 cm and 77.1% for pT > 1 cm (*p* = 0.006), adjusted hazard ratio 0.40 (0.16–0.99).

Another limitation arose as a result from the median year of BC diagnosis in our cohort being in the early 2000s, with hormone receptor status and especially HER2-status missing for a substantial proportion of cases. Therefore, we could not use TNBC as a variable for stratification. Instead, we used the imputed variables ER-status and HER2-status for adjustment. Further, the cause of death was unknown for a large proportion of cases, making BCSS analyses impossible. However, because we had a relatively young cohort with a median age of 42.3 years at BC diagnosis, and we censored at diagnosis of another cancer (including ovarian cancer), we can assume that the majority of the unknown causes of death are BC related. Finally, although a complete screening variable would be preferred, we expect our practical solution of a DNA-test result-based imputation provides a close enough approximation for our analysis on lymph node involvement where screening was taken into account.

Ultimately, we observed (1) better prognosis with a smaller tumor size at diagnosis, (2) possibly improved survival after adjuvant chemotherapy treatment for those with a pT1bN0 or pT1cN0 tumor, and (3) less lymph node involvement at diagnosis for those with a smaller tumor size. These findings confirm several potential benefits from intensive screening for women at high risk of developing BC due to a *BRCA1/2* mutation, under the assumption that screening indeed leads to finding BC when the tumor is small and before lymph node involvement occurs.

In conclusion, overall survival of *BRCA1/2-*associated breast cancer patients is better when they are diagnosed with a smaller tumor size within the pT1 category. Lymph node involvement is a frequent occurrence in *BRCA1/2-*associated BC and increases with larger tumor size. The results support current intensive screening strategies in *BRCA1/2* mutation carriers, aiming to detect preferentially pT1a/b BC to improve survival and reduce the need for systemic therapy. To achieve early detection more often, research into further optimization of imaging techniques may be warranted.

## Supplementary Information

Below is the link to the electronic supplementary material.

Electronic supplementary material 1 (DOC 36 kb)

## Data Availability

The data underlying this article are not publicly available due to the presence of highly sensitive patient information, but may be shared on reasonable request to the Hebon Steering Committee, when it does not conflict with Hebon policy or Dutch privacy law (GDPR).

## Abbreviations

BCC: Basal cell carcinoma of the skin
BC: Breast cancer
BCSS: Breast cancer-specific survival
CIN: Cervical intraepithelial neoplasia
CI: Confidence interval
ER: Estrogen receptor
HR: Hazard ratio
Hebon: Hereditary Breast and Ovarian Cancer Research Netherlands
HER2: Human Epidermal Growth Factor Receptor 2
ISH: In-situ hybridization
MRI: Magnetic Resonance Imaging
OS: Overall survival
PR: Progesterone receptor
RCT: Randomized controlled trial
RRM: Risk-reducing mastectomy
RRSO: Risk-reducing salpingo-oophorectomy
SCC: Squamous cell carcinoma of the skin
TNM: *TNM (Tumor Nodes Metastasis) classification **of malignant tumors*, UICC
TNBC: Triple-negative breast cancer
