# Supplementary material for: Survival of BRCA1/BRCA2-associated pT1 breast cancer patients, a cohort study
Source: Breast Cancer Res Treat. 2022 May 4;194(1):159–70. doi: 10.1007/s10549-022-06608-1 (PMC9167195; doi:10.1007/s10549-022-06608-1)
Supplement: Supplementary file 1 — Electronic supplementary material 1 (DOC 36 kb) [file 10549_2022_6608_MOESM1_ESM.doc]

**Supplemental materials**

*The multiple imputation model*

We used the Multiple Imputation by Chained Equations (MICE) method, as this allows for inclusion of different variable types (i.e. continuous, binary, categorical). We worked under the assumption of data missing at random (MAR), in some cases conditional on specific variables that were available. For example, the HER2 status variable was missing for 45% of our patients. However, 94,5% of missing were in the years 1990-2005, before HER2 status was routinely tested for and therefore expected to be missing. Variables included in the imputation model were those expected to be important for the Cox model (i.e. age at diagnosis, year of diagnosis, ER-status, HER2-status, tumor grade, chemotherapy treatment, endocrine treatment, tumor size, lymph node involvement and *BRCA* mutation), as well as auxiliary variables (those that correlate with the variables (or their missingness) that are to be imputed, providing a structure for the model to draw its imputations from) and the outcome variable (time to death) through the Nelson-Aalen cumulative hazard estimator, as described by White et al. (2009). Variables were imputed in ascending order of % missingness. Per imputed dataset, ten burn-in iterations were used.

We checked for performance of the imputation model, using traceplots for the variables with missing data and variance statistics using STATA’s build-in commands. Estimates were combined using Rubin’s Rules, as is standard with STATA’s “mi estimate”-command.

In descending order of %missing, we imputed the following variables: HER2-status (45% missing), PR-status (20.5% missing) ER-status (17.7% missing), Tumor grade (11.3% missing), endocrine therapy (1.6% missing). Potential auxiliary variables were selected based on correlation (r>0.1), association (p<0.1) or association with missingness of imputed variables. Ultimately, not all potential auxiliary variables were included in the MI model, if they caused collinearity or non-convergence of the model. The following auxiliary variables were used; tumor grade second BC, multifocality, PR-status, axillary lymph node diagnostics, second non-breast malignancy, timing of DNA test result. Fifty imputed datasets were generated.

| **Supplementary Table 1. Distribution of lymph node metastasis at diagnosis per pT1 category, stratified by *BRCA* mutation and time of DNA diagnosis (before or after BC diagnosis)** | | | | | | | | | | | | |
| --- | --- | --- | --- | --- | --- | --- | --- | --- | --- | --- | --- | --- |
| **Time of DNA diagnosis** | **Screened or DNA result before BC diagnosis (high probability of being screened) n=316 (33.3%)**  Overall pN+: 66/316 (20.9%) | | | | | | **Not screened or DNA result after BC diagnosis (low probability of being screened) n=634 (66.7%)**  Overall pN+: 205/634 (32.3%) | | | | | |
|  | **pT1a; n(%)** | | **pT1b; n(%)** | | **pT1c; n(%)** | | **pT1a; n(%)** | | **pT1b; n(%)** | | **pT1c; n(%)** | |
| Mutation | ***BRCA1*** | ***BRCA2*** | ***BRCA1*** | ***BRCA2*** | ***BRCA1*** | ***BRCA2*** | ***BRCA1*** | ***BRCA2*** | ***BRCA1*** | ***BRCA2*** | ***BRCA1*** | ***BRCA2*** |
| pN0 | 16 (94) | 16 (89) | 60 (86) | 31 (74) | 89 (78) | 38 (69) | 12 (75) | 7 (70) | 45 (79) | 23 (72) | 247 (72) | 95 (54) |
| pN+ | 1 (6) | 2 (11) | 10 (14) | 11 (26) | 25 (22) | 17 (31) | 4 (25) | 3 (30) | 12 (21) | 9 (28) | 97 (28) | 80 (46) |
| *BRCA1* vs *BRCA2* | **p=0.581** | | **p=0.118** | | **p=0.206** | | **p=0.780** | | **p=0.451** | | **p<0.001** | |
